# Excretion rates of 1,5-anhydro-D-glucitol, uric acid and microalbuminuria as glycemic control indexes in patients with type 2 diabetes

**DOI:** 10.1038/srep44291

**Published:** 2017-03-10

**Authors:** Cong Ma, Junqin Sheng, Zhiwen Liu, Minghao Guo

**Affiliations:** 1 Department of Endocrinology, Xuhui District Central Hospital, No. 966, Huaihai Zhong Road, Shanghai 200031, China; 2 Department of Nephrology, Xuhui District Central Hospital, No. 966, Huaihai Zhong Road, Shanghai 200031, China; 3 Department of Endocrinology, Shanghai Ninth People’s Hospital, Shanghai JiaoTong University School of Medicine, No. 639 Zhizaoju Road, Shanghai 200011, China

## Abstract

1,5-anhydroglucitol (1,5-AG), uric acid and urinary proteins are excreted into the urine with increasing glucosuria. In the present retrospective study we analyzed whether these factors could be used as indicators for type 2 diabetes mellitus (T2DM) glucose control in 6,766 (T2DM) patients. There were 3,988 cases (58.9%) with HbA1c ≤ 6.5%, 853 cases (12.61%) with HbA1c levels ranging from 6.5% to 7% and 1,925 cases (28.5%) with HbA1c > 7%. HbA1c percentages were correlated with age, MA and 1,5-AG serum concentrations (*P *<* *0.001). The serum uric acid concentration (*P *<* *0.001) was significantly lower in elevated MA (*P *<* *0.001) and 24-hour urinary protein (*P *=* *0.024) patients. Hb1Ac percentages (*P *<* *0.001) were significantly enhanced in patients with 1,5-AG serum concentrations ≤10 mg/L compared to >10 mg/L. With a derived receiver operating characteristic (ROC) curve, a 1,5-AG cut-off value of 11.55 mg/L for hyperglycemia could be diagnosed with a specificity of 71.2 (69.7–72.6) and a sensitivity of 75.3 (73.6–76.9). The serum 1,5-AG concentration is a marker for hyperglycemia and may be particularly useful as an indicator for short-term glycemic excursions in order to improve treatments in T2DM patients.

China has the highest incidence of diabetes worldwide with the prevalence increasing from 5.5% in 2001 to 9.7% in 2008 and to 11.6% in 2010, with an estimated number of 113.9 million diabetics^1^. Since diabetes does not present with obvious symptoms in the initial stages, laboratory diagnosis is essential to detect serum glucose changes, particularly in the early stages of diabetes and prediabetes conditions. The gold standard for detecting enhanced blood glucose concentrations is serum glucose measurements and the determination of HbA1c serum concentrations. HbA1c results from the glycation of hemoglobin in erythrocytes and indicates long-term (2–3 months) glycemia^2^. Alternative tests include the determination of fructosamine and glycated albumin levels, which are both formed via non-enzymatic bindings of blood glucose to serum proteins or albumin^3^,^4^. Glycated albumin and fructosamine serum concentrations decrease during the initial 2–3 weeks with half-times of 17.1 ± 2.8 and 12.2 ± 4.8 days, respectively^5^. As well as the effects of hyperglycemia on enhanced glycosylated serum proteins, the kidney re-absorption of 1,5-AG is also altered during hyperglycemic periods. 1,5-AG is a monosaccharide structurally similar to D-glucose and is derived mainly from dietary sources with a half-life of 1–2 weeks. It is not metabolized but excreted in the urine, with 99.9% being reabsorbed by the kidneys^6^,^7^. 1,5-AG levels are stable in healthy individuals independent of the prandial state, body weight or age^6^,^8^,^9^. However, when serum glucose levels exceed the threshold of glucosuria, reabsorption of 1,5-AG is competitively inhibited by glucose leading to a rapid reduction in serum levels. Therefore, reduced plasma 1,5-AG concentrations reflect episodes of hyperglycemia within 24 hours^10^,^11^,^12^. High blood glucose concentrations are also known to lead to elevated glomerular filtration rates in both diabetics and healthy subjects^13^. At present, the earliest clinical signals for diabetic nephropathy is the continuous increase in the urinary albumin excretion rate and the emergence of MA^14^. However, correlations between MA and blood glucose are not restricted to diabetic nephropathy patients, but have also been reported for pre-diabetes impaired fasting glucose cases^15^. One study has shown that controlling blood glucose concentrations in T2DM patients with gliclazide and pioglitazone could significantly reduce MA with similar improvements in blood glucose levels^16^. In contrast to MA, it has been proposed that similar to 1,5-AG, uric acid reabsorption in the proximal tubule is inhibited by hyperglycemia leading to decreased serum uric acid concentrations in diabetics^17^,^18^.

In the present study, we sought to evaluate correlations between MA and 24-hour urinary protein as well as HbA1c, serum uric acid and 1,5-AG concentrations in a large cohort of Chinese T2DM patients.

## Patients and Methods

The ethics committee of the hospital approved the study and written informed consent was obtained from all participants after discussion in Chinese aided by pictorial and written information. The study was conducted in accordance with the Declaration of Helsinki regarding ethical standards for research involving human subjects.

### Patients

In this retrospective study, we enrolled 6,766 patients who were diagnosed with T2DM and treated from January 2012 to June 2015 in the Xuhui District Central Hospital of Shanghai. The patients had been diagnosed as T2DM cases for more than 1 year by the 1999 WHO diagnostic criteria. Exclusion criteria were liver dysfunction, abnormal renal function (serum creatinine > 120 μmol/L), malignant tumor, hyperthyroidism or hypothyroidism, and hormone therapy. The treatment of the patients included diet control, oral hypoglycemic agents (sulfonylureas, biguanides or an α-glucosidase inhibitor) and insulin injections.

### Biochemical tests

We sampled fasting serum from patients, performed liver and kidney function tests, and tested the serum levels of 1,5-AG and HbA1c by high performance liquid chromatography tandem mass spectrometry (LC-MS/MS), (AP14000, Applied Biosystems Inc., USA).

TC, TG and uric acid concentrations were analyzed by enzymatic assay, whereas LDL-C and HDL-C was estimated calorimetrically using an automatic analyzer (ADV1A 2400, Siemens, German). MA and uric acid as well as 24-hour urine concentrations were measured by transmission turbidity using an automatic analyzer (BS-800M, Mindrayz, China).

The accuracy of low, medium and high concentration quality controls was within the 85–115% range, and the precision of CV was <15%. MA was defined as a urinary albumin concentration >30 mg/L^19^; hyperuricemia was defined as a uric acid concentration >420 μmol/L^20^.

### Statistical analysis

The statistical analyses were performed using SPSS for Windows (Version 13.0. Chicago, SPSS Inc.). All categorical variables are reported as frequencies. Continuous variables that were normally distributed are reported as the mean ± standard deviation. The comparison between continuous variables and categorical variables (such as gender) was analyzed by variance analysis and any relationship subsequently analyzed by linear-regression analysis. Multivariate analysis was performed using covariance analysis.

## Results

### Baseline and general condition of the patients

The 6,766 participants ranged from 21 to 101 years of age, with a mean of 74.18 ± 13.83 years. There were 3,503 male patients (48.2%) and 3,263 female patients (51.8%). 4,486 patients had hypertension, which was the main complication in 66.3% of the total patients, followed by coronary heart disease (CHD) with 1,600 cases (42.3%) and cerebral infarction (20.3%). The average value of HbA1c was 7.62 (ng/mL). It should be noted that 52.3% of the participants recruited in this study were elderly patients with diabetes (Table 1).

There were 3,988 cases (58.9%) with HbA1c ≤ 6.5%, 853 cases (12.61%) with HbA1c ranging from 6.5% to 7%, and 1,925 cases (28.5%) with HbA1c > 7%. It was shown that 58.9% of the elderly patients achieved an ideal standard value of HbA1c by drug control, 12.61% of the patients had relatively good control of blood glucose, but 28.5% of the patients had poor blood glucose control. Therefore, we observed the differences of serum 1,5-AG and uric acid as well as MA and 24-hour urine protein levels in the three HbA1c groups. We found that with an increase of HbA1c concentration, the serum 1,5-AG level significantly decreased and that MA levels were significantly increased. Both uric acid and 24-hour urinary protein showed no significant differences between the HbA1c ≤ 6.5%, 6.5~7% and >7% groups, respectively (Table 2).

Next, we divided the patients into two groups according to their 1.5–AG serum concentrations (1,5-AG ≤ 10 mg/L and 1,5-AG > 10 mg/L) and analyzed the correlations between MA, uric acid, HbA1c and 24-hour urinary protein levels in the two groups. We found that there were significant differences between MA, uric acid, HbA1c and 24-hour urinary protein levels in the 2 groups (Table 3).

**Figure 1:**
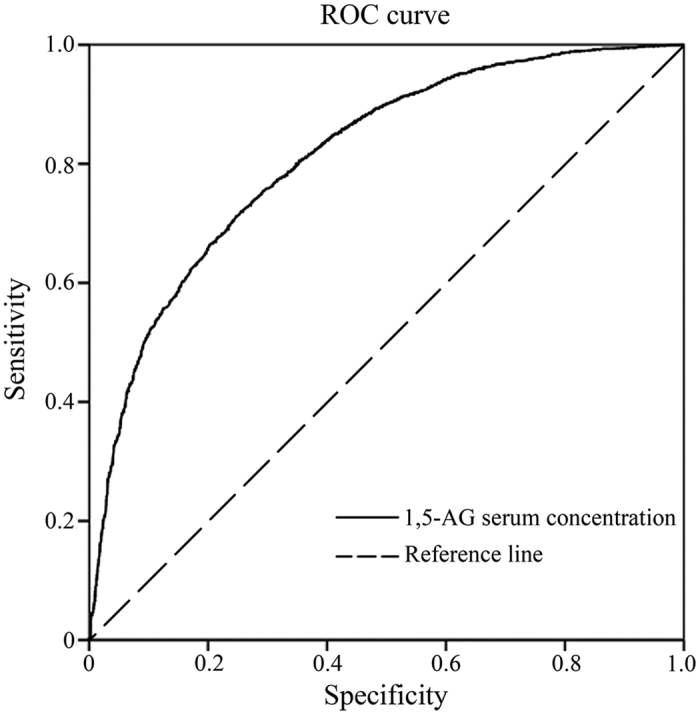
Sensitivity and specificity of 1,5-AG serum concentrations for prediction of hyperglycemia in a ROC curve.

Next, we performed receiver operating characteristic (ROC) curve analysis of plasma 1,5-AG. The sample of ROC analysis included some of the patients. The area of the ROC curve analysis for patients with diabetes was 0.813 (95% CI: 0.804–0.823). With a cut off point of 11.55 mg/L, the 1,5-AG serum concentration represented a sensitivity and specificity of 75.31% (95% CI: 73.7–76.9) and 71.15 (69.7–72.6) respectively, as diagnostic factor for diabetes (Fig. 1).

Finally, we compared 1,5-AG and uric acid serum concentrations as well as MA and 24-hour urinary protein concentrations as indicators for diabetes diagnosis and found that 1,5-AG was a relatively effective indicator of blood glucose control, with superior sensitivity and specificity than other indicators (Table 4).

## Discussion

HbA1c is a glycosylated hemoglobin test, which usually reflects the blood glucose control of patients over 8–12 weeks, so the results are stable and reliably reflect the average blood glucose levels within 120 days before a test. However, HbA1c is only slightly influenced by short-term glycemic excursions, such as postprandial hyperglycemia, whereas the concentration of 1,5-AG in the blood of diabetics reflects the short-term variations in blood glucose levels^6^. In a previous study, it was suggested that although the mean plasma glucose and HbA1c levels indicate good control, urinary glucose excretion might be intermittently high in insulin treated diabetes patients with concomitant low plasma 1–5 AG concentrations^21^. Another study demonstrated that 1,5-AG levels were not influenced by mild or moderate renal dysfunction in chronic kidney disease (CKD) stages 1–3^22^. Our study revealed that in patients with HbA1c percentages >7 the 1,5-AG serum concentrations were significantly lower, indicating that their hyperglycemia was probably not caused by a single event in the past, but most likely was the result of recent glycemic excursions. Different values have been reported for blood 1–5 AG concentrations in T2DM patients depending on the racial/ethnic groups, being higher in Asians and Africans compared to Caucasians^23^. However, our derived cut-off value of 11.55 mg/L is similar to that previously reported (10.7 mg/L^24^; 12 mg/L, ^12^14 mg/L^25^,^26^. The sensitivity of 75.3% and specificity of 71.2% in our measurements with a cut-off value of 11.55 mg/L 1,5-AG concentrations is in a similar range reported (78% and 72%) for predicting blood glucose enhancements of 2-hour post-challenge glucose ≥200 mg/dL in 75 g oral glucose tolerance tests with a 1–5 AG cut-off value of 14.2 μg/mL^27^. In the present study, we found when comparing the ≤10 mg/L 1,5-AG serum concentration group with the >10 mg/L 1,5-AG serum concentration group, that in the low 1,5 AG diabetics the serum uric acid concentrations were significantly lower, which supports the hypothesis that reabsorption of uric acid in the proximal tubule is inhibited by hyperglycemia^17^,^18^, because in the low 1,5-AG serum concentration group the HbA1c percentage was significantly higher. In contrast, in high 1,5-AG serum concentration patients, MA and 24-hour urinary protein were significantly reduced compared to the low 1,5-AG serum concentration patients. These data indicated that enhanced Hb1Ac values were accompanied by increased leakage of 1,5 AG, uric acid and proteins into the urine, which is in accordance with the previously published literature^11^,^16^,^28^. However, when comparing the sensitivities and specificities of MA and 24-hour urinary protein as well as uric acid serum concentrations as indicators for blood glucose control, the values were inferior compared to 1,5 AG levels.

The limitations of this study were the retrospective design in a single center with a high risk of selection bias. In addition, since we collected the data from 6,766 T2DM patients who were mostly from a local city that provided good economic and medical conditions, this study might not be representative of the entire country and larger studies including multiple collaborating centers will be warranted.

Our study confirmed that plasma 1,5-AG concentrations can reflect hyperglycemia and that the cut-off value in Chinese patients was 11.55 mg/L.

## Additional Information

**How to cite this article:** Ma, C. *et al*. Excretion rates of 1,5-anhydro-D-glucitol, uric acid and microalbuminuria as glycemic control indexes in patients with type 2 diabetes. *Sci. Rep.* **7**, 44291; doi: 10.1038/srep44291 (2017).

**Publisher's note:** Springer Nature remains neutral with regard to jurisdictional claims in published maps and institutional affiliations.

## Figures and Tables

**Table 1:** Demographic characteristics and levels of serum biochemical markers in patients with type-2 diabetes mellitus.

	Total number (N = 6,766)
Age (years)	74.18 (13.83)
≥65 years	52.3%
Gender	
Male	3,503 (51.8%)
Female	3,263 (48.2%)
Coronary heart disease	1,600 (42.3%)
Cerebrovascular disease	1,373 (20.3%)
Peripheral arterial disease	81.2 (1.2%)
Chronic kidney disease	602 (8.9%)
Hypertension	4,486 (66.3%)
TC (mmol/L) [Mean (SD)]	4.7 (1.5)
LDL-c (mmol/L) [Mean (SD)]	2.8 (1.2)
HDL-c (mmol/L) [Mean (SD)]	1.5 (0.7)
TG (mmol/L) [Mean (SD)]	2.0 (1.5)
Non HDL-c (mmol/L) [Mean (SD)]	3.5 (1.3)
Uric acid (μmol/L) [Mean (SD)]	320.33 (112.16)
Creatinine (μmol/L) [Mean (SD)]	83.6 (31.4)
MA (μg/mL) [Mean (SD)]	87.71 (82.53)
HbA1c (%) [Mean (SD)]	7.62 (1.66)
24-hour urinary protein (g/l) [Mean (SD)]	0.58 (1.29)
1,5-AG (mg/L) [Mean (SD)]	12.35 (8.09)

**Table 2:** Different HbA1c levels and their correlations with 1,5-AG, uric acid, MA and HbA1c percentages.

HbA1c	≤6.5	6.5%~7%	>7%	*P*-value
Total (n/%)	3,988 (58.9)	853 (12.61)	1,925 (28.5)	0.004
Age (years) (mean ± SD)	76.75 ± 14.25	75.44 ± 12.04	72.93 ± 13.15	<0.000
MA (μg/mL) (mean ± SD)	64.82 ± 68.39	72.64 ± 74.44	92.01 ± 84.13	<0.000
Uric acid (μmol/L) (mean ± SD)	342.94 ± 117.32	352.36 ± 93.5	328.12 ± 106.47	0.087
1,5-AG (mg/L) (mean ± SD)	18.3 ± 10.33	12.4 ± 7.52	6.35 ± 5.86	<0.000
24-h urinary protein (g/L) (mean ± SD)	0.55 ± 0.98	0.58 ± 1.46	0.48 ± 1.12	0.673

**Table 3:** Comparison of MA, Uric acid, HbA1c and 24-hour urinary protein in 1,5-AG ≤ 10 mg/L and 1,5-AG > 10 mg/L patient groups.

1,5-AG serum concentration (mg/L)	≤10 (N = 2,848)	>10 (N = 3,918)	*P*-value
Age (years) (mean ± SD)	74.18 ± 13.83	76.45 ± 13.66	<0.0001
MA (μg/mL) (mean ± SD)	87.71 ± 82.53	62.91 ± 66.67	<0.0001
Uric acid (μmol/L) (mean ± SD)	320.33 ± 112.16	353.97 ± 109.99	<0.0001
HbA1c% (mean ± SD)	7.62 ± 1.66	6.17 ± 0.73	<0.0001
24-hour urinary protein (g/L) (mean ± SD)	0.58 ± 1.29	0.40 ± 0.77	0.0244

**Table 4:** Sensitivity and specificity of 1,5-AG and uric acid serum concentrations as well as MA and 24-hour urinary protein as indicators for blood glucose control.

	1,5-AG ≤ 11.55 mg/L, 95% CI	MA ≥ 30 (μg/mL) 95% CI	Uric acid ≥ 420 (μmol/L), 95% CI	24-hour urinary protein ≥ 0.15 g/L (g/l), 95% CI
Sensitivity	75.3 (73.6–76.9)	66.2 (63.8–68.5)	20.5 (16.2–25.3)	51.4 (46.9–55.9)
Specificity	71.2 (69.7–72.6)	42.8 (40.7–44.8)	77.9 (74.2–81.3)	54.4 (47.1–61.6)
